# Improving Physician Performance Through Internet-Based Interventions: Who Will Participate?

**DOI:** 10.2196/jmir.7.4.e48

**Published:** 2005-09-02

**Authors:** Terry C Wall, M Anwarul Huq Mian, Midge N Ray, Linda Casebeer, Blanche C Collins, Catarina I Kiefe, Norman Weissman, Jeroan J Allison

**Affiliations:** ^1^Department of PediatricsUniversity of Alabama at BirminghamBirminghamALUSA; ^2^Center for Outcomes and Effectiveness Research and EducationUniversity of Alabama at BirminghamBirminghamALUSA; ^3^School of Health Related ProfessionsUniversity of Alabama at BirminghamBirminghamALUSA; ^4^Department of Health Services AdministrationUniversity of Alabama at BirminghamBirminghamALUSA; ^5^Division of Continuing Medical EducationUniversity of Alabama at BirminghamBirminghamALUSA; ^6^Department of MedicineUniversity of Alabama at BirminghamBirminghamALUSA; ^7^Birmingham Veterans Affairs Medical CenterBirminghamALUSA

**Keywords:** Physician recruitment, Chlamydia, chlamydiosis, Internet-based interventions, continuing medical education

## Abstract

**Background:**

The availability of Internet-based continuing medical education is rapidly increasing, but little is known about recruitment of physicians to these interventions.

**Objective:**

The purpose of this study was to examine predictors of physician participation in an Internet intervention designed to increase screening of young women at risk for chlamydiosis.

**Methods:**

Eligibility was based on administrative claims data, and eligible physicians received recruitment letters via fax and/or courier. Recruited offices had at least one physician who agreed to participate in the study by providing an email address. After one physician from an office was recruited, intensive recruitment of that office ceased. Email messages reminded individual physicians to participate by logging on to the Internet site.

**Results:**

Of the eligible offices, 325 (33.2%) were recruited, from which 207 physicians (52.8%) participated. Recruited versus nonrecruited offices had more eligible patients (mean number of eligible patients per office: 44.1 vs 33.6; *P* < .001), more eligible physicians (mean number of eligible physicians per office: 6.2 vs 4.1; *P* < .001), and fewer doctors of osteopathy (mean percent of eligible physicians per office who were doctors of osteopathy: 20.5% vs 26.4%; *P* = .02). Multivariable analysis revealed that the odds of recruiting at least one physician from an office were greater if the office had more eligible patients and more eligible physicians. More participating versus nonparticipating physicians were female (mean percent of female recruited physicians: 39.1% vs 27.0%; *P* = .01); fewer participating physicians were doctors of osteopathy (mean percent of recruited physicians who were doctors of osteopathy: 15.5% vs 23.9%; *P* = .04) or international medical graduates (mean percent of recruited physicians who were international graduates: 12.3% vs 23.8%; *P* = .003). Multivariable analysis revealed that the odds of a physician participating were greater if the physician was older than 55 years (OR = 2.31; 95% CI = 1.09–4.93) and was from an office with a higher *Chlamydia* screening rate in the upper tertile (OR = 2.26; 95% CI = 1.23–4.16).

**Conclusions:**

Physician participation in an Internet continuing medical education intervention varied significantly by physician and office characteristics.

## Introduction

Many interventions, including guidelines and educational programs, have been designed to improve quality of medical care, but engaging physicians in these interventions can be challenging. Although traditional continuing medical education (CME) courses tend to have little impact on physician behavior, they remain a popular form of continuing education as physicians attempt to stay current with medical practice and meet mandated CME requirements for licensure and certification [1,2]. However, traditional CME courses can be difficult to accommodate into physicians’ busy schedules [3]. These time constraints, as well as other factors, have led to the development of alternative methods, such as Internet-based CME—a form that is increasingly being used to reach physicians and which has the potential to reach large audiences.

Improving quality of care has historically been a major goal of CME activities. However, low rates of physician participation may bias results when such interventions are applied to change physician behavior in clinical settings [4]. When used as broad interventions to change behavior, even well-designed interventions may have limited impact as a result of low participation. Internet-based interventions hold the promise of increasing access to, and participation in, CME.

For Internet-based interventions to be effective they must reach their target audience. Understanding participation patterns and barriers to participation will help advance this important delivery mode of continuing education and improve quality.

We conducted a study to measure and improve *Chlamydia* screening rates among primary care physicians through a randomized controlled trial testing the use of an Internet-based, physician-targeted CME intervention that incorporated educational modules and provider audit and feedback. The purpose of this study was to identify potential physician and office characteristics that might predict physician participation in future Internet-based CME interventions.

## Methods

We conducted a retrospective analysis of data generated from the randomized trial “An Internet Intervention to Promote *Chlamydia* Screening.” Study recruitment proceeded in two phases: phase I focused on the primary care office, and phase II targeted individual physicians from recruited offices. In the analysis of phase I, we examined factors associated with office recruitment. In the analysis of phase II, we examined factors associated with physician participation.

This study was approved by the Institutional Review Boards of the University of Alabama at Birmingham and the managed care organization.

### Overview of Parent Study

The parent study, funded by the Agency for Healthcare Research and Quality as part of the second Translating Research into Practice initiative, tested an Internet intervention for primary care physicians and was performed in collaboration with a large, national managed care organization. The intervention was designed to increase *Chlamydia* screening of at-risk, young women by primary care physicians.

A series of Internet CME modules focusing on chlamydiosis were developed for the intervention. The goal of the instructional design of the online program was to create a multifaceted and multiphase online physician intervention based on current evidence of what is effective in CME. Delivery was via an asynchronous mode: physicians could log on at any time to participate. Email announcements and reminders with direct course links were used to alert physicians to the introduction of the course as well as three updates. Four separate modules were introduced quarterly. Components of the modules included (1) interactive unfolding cases with branching pathways designed to provide remediation based on the physician’s response to the case, (2) a quality improvement toolbox with resources to support office improvements in *Chlamydia screening*, (3) feedback to embedded questions so that participants could compare their responses to those of their peers, and (4) feedback of data on *Chlamydia* screening from the practice compared to peers within the overall group of practitioners. No online discussions were included. The intervention was designed and developed with Dreamweaver software (Dreamweaver MX, Macromedia, Inc., San Francisco, CA, USA) and used a SQL server database.

The Internet-based intervention for the control group was described to the participants as a CME course on women’s health for primary care physicians. Four modules, one each quarter, were offered to participants, and physicians could log on at any time to participate. The modules focused on women’s health issues unrelated to *Chlamydia* screening and included cardiovascular health and prevention of osteoporosis. The modules were text only and required participants to complete a post-test for CME credit. One category 1 CME credit was offered for each module. There was no mechanism for online discussions.

The intervention was designed for primary care physicians from internal medicine, family medicine/general practice, and pediatrics. Internists and pediatricians with a subspecialty board certification were not eligible. Physicians were randomized to an intervention or control group upon first logging on to the study Internet site. After one physician from a given office was randomized, all other physicians from the same office were assigned to the same study group.

*Chlamydia* screening rates, calculated by the managed care organization for each office, were based upon criteria from the Health Employers Data and Information Set (HEDIS) and provided the main outcomes for the parent study. The denominators for the rates were at-risk women identified from the 2001 HEDIS Technical Specifications applied to administrative data in the calendar year 2000. The HEDIS specifications were designed to identify women between the ages of 16 and 26 years who, based on health care services reflected in administrative data, were sexually active and therefore at risk for *Chlamydia* infections. HEDIS measures used pharmacy data (NDC codes) and claims/encounter data (ICD-9-CM and CPT-4 codes) to identify these health care services, which included pregnancy-related services, contraceptive prescriptions, screening for cervical cancer, and sexually transmitted diseases. The numerator was the number of women in the denominator who had claims data evidence of laboratory testing for *Chlamydia* during the baseline calendar year.

### Recruitment and Enrollment

Phase I recruitment occurred at the office level. Each eligible primary care office had at least 20 patients aged 16 to 26 years who were at risk for chlamydiosis based on HEDIS criteria. In November 2001, all potentially eligible physicians (n = 4673) in eligible offices (n = 978) were faxed recruitment letters (Multimedia Appendix 1) inviting participation in the study. Recruitment letters were faxed twice. Initial nonresponders were then sent invitations by courier, but not if another physician from the same office had already been recruited. Letters described the project in general terms, as an Internet-based intervention to improve the care that physicians deliver to their female patients, but it did not indicate *Chlamydia* screening rates as the focus. Because the main purpose of phase I recruitment was to maximize the number of recruited offices, when at least one physician from an office agreed to participate, that office was labeled as “recruited” and no additional recruitment effort was made.

In phase II of recruitment, all physicians who provided their email address in phase I were invited to log on to the study Internet site. The intervention was initiated in February 2002 with an email broadcast to all recruited offices. Emails contained the website address, which connected directly with the module. Recruited physicians received email reminders (Multimedia Appendix 2) monthly and then weekly for a total of up to 33 reminders over a 45-week period until they logged on or asked to be dropped from the study. Only 2 physicians withdrew, asking not to receive additional emails. Emails for 18 physicians were returned because of invalid email addresses, and 3 email addresses did not belong to physicians. Intervention and control group physicians received email reminders according to the same protocol.

### Data Sources

For all analyses, study variables were either (1) measured at the office level (patients/office and physicians/office), (2) measured at the patient level but available only at the office level (*Chlamydia* screening rates), or (3) measured at the physician level (physician age, gender, ethnicity, type of degree, country of graduation from medical school). Office characteristics were obtained from managed care organization administrative data. *Chlamydia* screening rates were calculated at the office level by the managed care organization based on HEDIS specifications. Other office characteristics were derived from managed care organization administrative data, and physician characteristics were derived from the American Medical Association’s physician master file.

### Analyses

The analysis for phase I examined factors associated with office recruitment (Tables 1 and 2) among all eligible offices (n = 978), and the analysis for phase II examined factors associated with physician participation (Tables 3 and 4) among all recruited physicians (n = 392). An office was labeled as recruited if at least one physician from that office was recruited. Physicians were defined as recruited if they provided an email address for subsequent contact. Participation was defined as having logged on to the study Internet site, regardless of how much of the material was completed. For phase I office-level analyses, the outcome was a dichotomous variable indicating whether the office had been recruited. For phase II physician-level analyses, the outcome was a dichotomous variable indicating whether the physician participated in the intervention. Independent variables included office and physician characteristics as described above.

Statistical significance was assessed using the chi-square statistic for categorical variables and the ANOVA for continuous variables for the bivariate analyses (Tables 1 and 3). Logistic regression was used for the phase I multivariable analysis (Table 2). For the phase II multivariable analysis, generalized estimation equations with a logit link accounted for the clustering of physicians within offices (Table 4). Because we were mainly interested in examining the independent contribution of covariates to either office recruitment or physician participation, we did not engage in covariate selection exercises to optimize the predictive power of the multivariable models. Instead, two models were constructed, each containing all important covariates.

## Results

Of the 978 eligible offices, 325 (33.2%) were recruited by having at least one physician agree to participate. Overall, eligible offices had an average of 4.8 eligible primary care physicians and 39.1 female patients considered at risk for chlamydiosis. The average screening rate was 16.2%. Of the 392 recruited physicians, 207 (52.8%) participated in the intervention. Eligible physicians were, on average, 44.4 years old and represented 25 US states. About one third of the physicians were female (33.4%), and most were white (82.3%). Most physicians were in internal medicine (36.0%) or family practice (52.3%); fewer (11.7%) were in pediatrics. There was a significant representation of doctors of osteopathy (19.5%) and international medical graduates (17.8%).

Recruited versus nonrecruited offices had more eligible patients (mean number of eligible patients per office: 44.1 vs 33.6; *P* < .001) and physicians (mean number of eligible physicians per office: 6.2 vs 4.1; *P* < .001) (Table 1). Recruited offices also had more family practice physicians and fewer pediatricians, as well as fewer doctors of osteopathy. However, in the multivariable analyses, only the number of eligible patients and physicians remained significant independent predictors of office recruitment status (Table 2). The odds of an office being recruited were greater if the number of eligible patients was in the top 10% for all offices and the number of eligible physicians was in the top 10% for all offices.

**Table 1:** Characteristics of eligible primary care offices[Table-fn table1fn1] and eligible physicians from a large managed care organization, by office recruitment status[Table-fn table1fn2]

		**Recruited(n = 325)**	**NotRecruited(n = 653)**	***P* value**
**Office characteristics**				
Eligible patients, mean		44.1	36.6	< .001
*Chlamydia* screening rate, mean*		16.3	16.2	.88
Eligible physicians, mean		6.2	4.1	< .001
Physician characteristics**				
Age, mean (years)		44.3	44.4	.90
Female physicians, mean (%)		33.7	33.7	.99
	***Ethnicity†***			
	White, mean (%)	81.7	79.6	.36
	African American, mean (%)	4.5	6.7	.12
	Asian, mean (%)	9.8	9.3	.76
	Hispanic, mean (%)	3.9	4.4	.68
	***Specialty***			
	Internal medicine, mean (%)	36.4	34.2	.45
	Family medicine/general practice, mean (%)	52.9	58.4	.08
	Pediatrics, mean (%)	10.7	7.5	.07
Doctor of osteopathy, mean (%)		20.5	26.4	.02
International medical graduate, mean (%)		19.5	18.8	.75

Eligible offices had at least 1 eligible physician with at least 20 female patients who were candidates for *Chlamydia* screening according to the HEDIS Technical Specifications, 2000.

Recruited offices had at least 1 physician provide an email address for subsequent contact.

^*^ *Chlamydia* screening rates were determined from HEDIS Technical Specifications, 2000.

^**^ Physician characteristics were reported at office level as unweighted averages across all offices.

^†^ This information was missing for 30.0% of the physicians in the sample.

**Table 2:** Multivariable logistic model for primary care office recruitment[Table-fn table2fn1] among all eligible primary care offices (n = 821; c statistic = 0.622)[Table-fn table2fn2]

		**Odds Ratio**	**95% Confidence Interval**	
No. eligible patients ≥ 90th percentile*		2.68	1.67	4.31
*Chlamydia* screening rate**				
	Lower tertile	-	-	-
	Middle tertile	1.09	0.77	1.56
	Upper tertile	0.94	0.66	1.36
No. eligible physicians ≥ 90th percentile†		1.93	1.23	3.03
**Physicians**‡				
Age, mean (years)		1.01	0.99	1.03
Female, mean (%)		0.83	0.51	1.36
	***Ethnicity***			
	White, mean (%)	-	-	-
	African American, mean (%)	0.61	0.27	1.40
	Asian, mean (%)	1.01	0.50	2.05
	Hispanic, mean (%)	0.79	0.30	2.07
	***Specialty***			
	Internal medicine, mean (%)	-	-	-
	Family medicine/general practice, mean (%)	1.12	0.78	1.62
	Pediatrics, mean (%)	1.56	0.87	2.83
Doctor of osteopathy, mean (%)		1.04	0.63	1.71
International medical graduate, mean (%)		1.33	0.75	2.36

Recruited offices had at least 1 physician provide an email address for subsequent contact.

Eligible offices had at least 1 eligible physician with at least 20 female patients who were candidates for *Chlamydia* screening according to HEDIS Technical Specifications, 2000. The number is reduced due to missing data.

^*^ Dichotomous variable indicating whether number of eligible patients in office was ≥ 90th percentile for number of eligible patients in all offices.

^**^ *Chlamydia* screening rates were determined from HEDIS Technical Specifications, 2000.

^†^ Dichotomous variable indicating whether number of eligible physicians in office was ≥ 90th percentile for number of eligible physicians in all offices.

^‡^ Physician characteristics were summarized at office level as unweighted averages across all offices. Odds represent one-unit increase.

Participating versus nonparticipating physicians were more likely to be female (mean percent of female recruited physicians: 39.1% vs 27.0%; *P* = .01) and less likely to be doctors of osteopathy (mean percent of recruited physicians who were doctors of osteopathy: 15.5% vs 23.9%; *P* = .04) or international medical school graduates ((mean percent of recruited physicians who were international graduates: 12.3% vs 23.8%; *P* = .003) (Table 3). From the multivariable analysis, being from an office with *Chlamydia* screening rates in the top tertile was associated with greater odds of participation. Also, physicians older than 55 years were more likely to participate (Table 4).

**Table 3:** Characteristics of 392 primary care recruited physicians[Table-fn table3fn1] from a large managed care organization, by physician participation status[Table-fn table3fn2]

		**Participated(n = 207)**	**Did NotParticipate(n = 185)**	***P* value**
Age, mean (years)		44.9	43.8	.22
Female, mean (%)		39.1	27.0	.01
	Ethnicity*			
	White, mean (%)	82.1	82.6	.91
	African American, mean (%)	4.5	2.3	.29
	Asian, mean (%)	9.5	10.6	.73
	Hispanic, mean (%)	4.0	4.6	.80
	***Specialty***			
	Internal medicine, mean (%)	36.7	35.1	.75
	Family medicine/general practice, mean (%)	53.6	50.8	.58
	Pediatrics, mean (%)	9.7	14.1	.18
Doctor of osteopathy, mean (%)		15.5	23.9	.04
International medical graduate, mean (%)		12.3	23.8	.003

Recruited physicians provided their email address for subsequent contact.

Participating physicians logged on to the study Internet site.

^*^ This information was missing for 30.0% of the physicians in the sample.

**Table 4:** Multivariable logistic model[Table-fn table4fn1] for primary care physician participation among all recruited physicians (n = 324)[Table-fn table4fn2]

		**Odds Ratio**	**95% Confidence Interval**	
**Office characteristics**				
No. eligible patients ≥ 90th percentile*		0.55	0.21	1.42
*Chlamydia* screening rate**				
	Lower tertile	-	-	-
	Middle tertile	1.29	0.73	2.31
	Upper tertile	2.26	1.23	4.16
No. eligible physicians per office ≥ 90th percentile†		1.46	0.66	3.22
**Physician characteristics**				
Age > 55 years		2.31	1.09	4.93
Female, mean (%)		1.57	0.92	2.70
	***Ethnicity***			
	White, mean (%)	-	-	-
	African American, mean (%)	1.82	0.43	7.71
	Asian, mean (%)	0.85	0.35	2.02
	Hispanic, mean (%)	1.22	0.43	3.45
	***Specialty***			
	Internal medicine, mean (%)	-	-	-
	Family medicine/general practice, mean (%)	1.09	0.64	1.85
	Pediatrics, mean (%)	0.46	0.20	1.03
Doctor of osteopathy, mean (%)		0.65	0.33	1.28
International medical graduate, mean (%)		0.57	0.28	1.16

Based on generalized estimation equations with logit link accounting for clustering of physicians within offices.

Recruited physicians provided their email address for subsequent contact. Participating physicians logged on to the study Internet site. The number is reduced due to missing data.

^*^ Dichotomous variable indicating whether number of eligible patients for a given office was ≥ 90th percentile for number of eligible patients for all offices. Patient eligibility for *Chlamydia* screening was defined by HEDIS Technical Specifications, 2000.

^**^ Offices classified according to *Chlamydia* screening rate tertiles. *Chlamydia* screening rates were determined from HEDIS Technical Specifications, 2000.

^†^ Dichotomous variable indicating whether number of physicians in the office of primary care physician was ≥ 90th percentile for number of physicians in all offices. Eligible physicians had at least 20 eligible female patients.

## Discussion

Our low-intensity recruitment methods, including fax and courier delivery and email reminders, allowed us to meet the recruitment goal of approximately 200 offices. Using these methods, we were able to recruit a geographically diverse sample of physicians who were not affiliated with our institution or research team. Our study is unique in that it provides a detailed description of predictors of physician recruitment and subsequent participation in an Internet-based intervention to improve care.

### Recruiting Physicians for Office-Based Research

Many methods have been used to recruit physicians for office-based clinical research. The most intensive approach involves physician-to-physician contact, either by telephone or in person at the practice site. Initial contact by mail is commonly used, either alone or in conjunction with other methods. Less intensive approaches include contact by fax or email. Recruitment that combines several approaches will probably produce a higher participation rate, but the intervention team must determine if the higher participation rate justifies the added investment [5,6].

McBride et al compared three methods, based on point of contact, for recruiting community primary care physicians in a preventive services clinical trial: direct to primary care physicians, through the health maintenance organization to practice leaders, or direct to practice leaders [5]. All three methods involved an initial mailing, either from the university or the health maintenance organization, as well as follow-up phone calls and an informational on-site meeting with the practice. Outcomes included response rates, participation rates, and comparative costs of each method. Of the 86 eligible practices, 52 (60%) agreed to participate. Mailings to individual physicians were the least efficient means of recruiting, while targeting medical directors was the most efficient method in this trial.

While some physicians will enroll after only minimal efforts are expended to recruit them, others will require more intense recruitment efforts. Achieving large numbers of recruited subjects for minimal costs has obvious benefits, allowing more intensive and expensive efforts to be focused on those individuals that are more difficult to recruit. Having a staged approach to recruitment conserves valuable resources.

We met our recruitment goal of at least one physician from approximately 200 offices without using intensive recruitment methods. Therefore, we feel that this study underestimates the true percentage of physicians willing to engage in an Internet intervention. In addition, we did not ask participating physicians to recruit others from the same office, although such a strategy may prove useful for future studies.

Participation in projects targeting physicians may also be affected by physician characteristics. Shelton et al studied recruitment and retention of community primary care practices in a study to improve cancer screening and counseling [7]. Their initial decline rate was only 6%, but the refusal rate reached 30% by the time the intervention was implemented. Study participants were more often younger, located in rural areas, and family practitioners rather than internists.

### Recruiting Physicians for CME Studies

Even though this was a research study, the physicians’ viewed the study primarily as an opportunity to participate in a CME activity to improve care. Our intervention involved both an Internet-based CME component and physician feedback. While we could not find another study examining physician recruitment and participation in a similarly designed intervention, there are several studies of physician participation in traditional CME courses [3,8-12]. Factors which influence physician participation in traditional CME courses include licensure requirements and opportunities for review or general updates and for interaction with colleagues, especially in the context of professional societies [8,9]. Internet-based CME may be tailored to meet individual needs and be more interactive than traditional CME.

Goulet et al found that being older, having a rural practice, and having a solo practice were associated with less participation in group CME activities [10]. Distance to a CME activity may be more of an issue for rural physicians, while being in solo practice would significantly limit available time. Gerbert et al reported their experience with a study that used traditional CME to improve outpatient management of chronic obstructive pulmonary disease [11]. Of 2600 eligible physicians invited to participate, 277 (11%) declined. Of the 171 (7%) who expressed initial interest in participating, only 89 (3%) enrolled and only 63 (2%) actually participated. Board-certified physicians and family practitioners were more likely to participate.

In our study, we are able to distinguish recruitment and participation as distinct steps and to examine how these processes may be influenced by office and physician characteristics. Because our goal was to recruit the required number of offices and because we increased the intensity of our recruitment efforts for offices that did not initially respond, we cannot draw conclusions about the influence of practice type (group vs solo) on recruitment. Recruitment was more common from offices with more physicians, which is likely a direct effect of our recruitment strategy. Recruitment was also more common from offices that had a greater number of eligible female patients, probably reflecting the physician’s perceived relevance for the CME program.

Different associations were found when physician-level participation after successful recruitment was examined. Practice composition based on physician gender, educational track, and international training was associated with physician participation only in the bivariate analyses. In the multivariable analysis, being an older physician and being in the highest *Chlamydia* screening group predicted participation. We did not expect older physicians to be more likely to participate in an Internet-based intervention. Older physicians are less likely to participate in group CME and more likely to obtain CME through independent reading and associated CME credits [10]. Participation in Internet-based CME may have a similar pattern in that it can be done at one’s convenience and without time away from practice and family.

Barriers to participation in traditional CME include time away from practice and family, costs of travel, and lack of relevance of general topics to specific patient problems [3,12]. Theoretically, Internet-based CME overcomes some of these barriers in that it is available to any physician with Internet access, not being affected by geographic location. In addition, Internet-based CME can be accessed at any time, making it possible for busy physicians to participate without restricting patient appointments. Recent surveys have shown that virtually all physicians have access to the Internet at work or at home [13].

CME is increasingly more available via the Internet, making it much more accessible than traditional CME activities. In addition, use of Web-based technologies is expected to increase [8,14]. Understanding physician factors associated with participation will assist in designing future recruitment efforts as well as Internet-based interventions.

### Limitations

Our study was limited to physicians with email access who met the specific inclusion criteria of being a member in a specified health maintenance organization–based provider network, practicing in one of the designated specialties (internal medicine, family practice, or pediatrics), and practicing in an office caring for a minimum number of at-risk women. Since we do not know the number of physicians with email, we do not know the true number of eligible physicians. Our recruitment methods were meant to be minimally intrusive, but we do not know who actually received the faxes and emails and made the decision of whether to participate. Because our study was not designed to maximally recruit physicians and determine response rates, we do not know what the response would have been to more intense recruitment efforts. Since we had a low rate of participation from international medical graduates, our results may not be generalized to this group. Special efforts may be needed in future projects in order to achieve greater participation from international medical graduates.

### Conclusions

Recruiting physicians for participation in projects for practice improvement is a challenging process which requires multiple contact points and may require multiple modalities. Using a staged approach to recruitment saves valuable resources for recruiting those physicians who are more difficult to recruit.

In addition, recruitment does not equate with participation. Our initial contact was via mail from the managed care organization followed by contact from the study team via fax. However, many physicians received repeated emails before actually participating in the study. Fortunately, our study was designed to use email reminders as a means of contact following actual recruitment. Use of other methodologies could have resulted in unpredictably high costs for continued mailings, courier deliveries, or phone contacts.

Ideally, research studies should recruit a diverse population of participants that will represent the population from which the sample is drawn. However, multiple studies have shown that physician factors may play a role in participation in research studies. Understanding the role of these factors may help in the design of the recruitment process. While we found some physician and practice factors to be associated with participation, many were not. This suggests that our recruitment efforts resulted in a sample that was reasonably representative of the larger population.

## Multimedia Appendix 1

Recruitment letter.

## Multimedia Appendix 2

Email reminder.

## Abbreviations

CME: continuing medical education
HEDIS: Health Employers Data and Information Set
