# Supplementary material for: Improving Physician Performance Through Internet-Based Interventions: Who Will Participate?
Source: J Med Internet Res. 2005 Sep 2;7(4):e48. doi: 10.2196/jmir.7.4.e48 (PMC1550676; doi:10.2196/jmir.7.4.e48)
Supplement: Supplementary file 1 [file jmir_v7i4e48_app1.pdf]

SCHOOL OF
MEDICINE
Division of Continuing Medical Education

November 26, 2001

Dear Dr.

Aetna U.S. Healthcare recently sent you an invitation to participate in a collaborative project sponsored by Aetna and the University of Alabama School of Medicine involving a series of primary care Continuing Medical Education (CME) courses. This research project, funded by the Agency for Healthcare Research and Quality, provides an excellent opportunity for you to assess your knowledge of primary care issues over the Internet in an innovative and interactive manner. Routinely collected Aetna data may be used to customize the Internet modules to the individual physician. The benefits of participating include:

- Access to cases relevant to your practice
- 4 hours of Category 1 CME credit at no cost
- Courses can be taken at your convenience, 24 hours a day 7 days a week
- Instant online credit certificate for successful completion of the course

Your participation involves the completion of four courses offered over twelve months beginning January 1, 2002 and a short post-assessment survey. The time commitment is about 30 minutes per course and about 15 minutes at the end of the project to complete a post-assessment survey. You must have Internet access to participate in these courses.

To register for this series of online CME courses, please complete and return the registration form below by fax to: Division of CME, University of Alabama School of Medicine, Fax: 205-934-1939.

Thank you for your consideration of this exciting program.

Sincerely,

Linda Casebeer, PhD
Associate Director

Name ....

Address ....

....

Email ....

1521 Building
1521 11th Avenue South
205.934.2687
205.934.1939
www.uab.edu/cme

The University of
Alabama at Birmingham
Mailing Address:
1521 BLDG
1530 3RD AVE S.
