# Supplementary material for: Improving Physician Performance Through Internet-Based Interventions: Who Will Participate?
Source: J Med Internet Res. 2005 Sep 2;7(4):e48. doi: 10.2196/jmir.7.4.e48 (PMC1550676; doi:10.2196/jmir.7.4.e48)
Supplement: Supplementary file 2 [file jmir_v7i4e48_app2.pdf]

From: "UAB Division of CME" <cme@uab.edu>
To: <email address>
Subject: New online women's health module ready for your review
Date: Wed, 4 Sep 2002 11:14:40 -0500

Dear Dr.

Thank you for participating in the University of Alabama School of Medicine Division of CME Online Women's Health Courses. A second module is now ready for your review and can be accessed at the following url:

http://www-cme.erep.uab.edu/aetna/module2/index.html

Upon successful completion of the course, you may earn an additional hour of Category 1 CME credit at no charge and access to an immediate online printable certificate. We look forward to seeing you online.

Linda Casebeer, Ph.D.
Associate Director
Division of Continuing Medical Education
University of Alabama School of Medicine
